# Editorial: Systems Immunology and Translational Research in Infectious Diseases

**DOI:** 10.3389/fimmu.2026.1791482

**Published:** 2026-01-28

**Authors:** Mariana Araújo-Pereira, Caian L. Vinhaes, Artur T. L. Queiroz, Bruno B. Andrade

**Affiliations:** 1 Laboratory of Clinical and Translational Research, Gonçalo Moniz Institute, Oswaldo Cruz, Foundation, Salvador, Brazil; 2 The Sybil Lab, MONSTER Institute for Education, Care, Research, and Technological Development in Health, Salvador, Brazil; 3 Clinical Research Laboratory on Mycobacteria (LAPCLIN-TB), Evandro Chagas National Institute of Infectious Diseases, Oswaldo Cruz Foundation, Rio de Janeiro, Brazil; 4 The Genios Lab, MONSTER Institute for Education, Care, Research, and Technological Development in Health, Salvador, Brazil; 5 Division of Infectious Diseases, Department of Medicine, Johns Hopkins University, Baltimore, MD, United States; 6 Department of International Health, Bloomberg School of Public Health, Johns Hopkins University, Baltimore, MD, United States; 7 The Bright Lab, MONSTER Institute for Education, Care, Research, and Technological Development in Health, Salvador, Brazil

**Keywords:** infectious diseases, omics, systems biology, systems immunology, translational medicine

The immune response to infection is a dynamic and multilayered process, shaped by host factors, such as genetic background and comorbidities, pathogen burden, and environmental factors (1, 2). While reductionist, hypothesis-driven studies, typically centered on one mediator, one pathway, or one cell type at a time, have provided critical insights, they often fall short of capturing the complexity and heterogeneity of immune responses across infectious diseases.

Systems immunology, by integrating high-dimensional datasets with clinical phenotypes, offers a comprehensive framework to identify mechanistic patterns, predictive signatures, and therapeutic targets (3, 4). This Research Topic brings together nine original studies that illustrate how systems-based approaches are advancing translational research in infectious diseases (from diagnostic tools and prognostic models to the development of novel vaccines) reaffirming the centrality of integrated immunological analysis in modern biotechnology and biomedicine.

The works explore large datasets (transcriptomes, serological panels, inflammatory indices, gene signatures, and clinical markers) to redefine risk stratification, differential diagnosis, and target discovery in acute, chronic, and post-infectious conditions. This approach encompassing sepsis, viral and bacterial respiratory infections, HIV-TB, acute appendicitis, Lyme disease, and long COVID-19, illustrates the transition from describing inflammatory phenotypes to predictive models and personalized interventions - in several infectious scenarios, a central goal of truly translational research.

Across these contributions, a consistent theme emerges: the recontextualization of immune data within real-world clinical settings. Zhu et al., for instance, evaluated pediatric patients with acute respiratory infections, quantifying myxovirus resistance protein A (MxA) to distinguish viral from bacterial etiologies. In an age group where symptom-based diagnosis is often hard and imprecise, the study supports the utility of MxA as a bedside tool to guide antimicrobial decisions, with potential application as an antimicrobial stewardship tool. Similarly, focused on differential diagnosis, Zhang et al. analyzed serological responses in patients with Lyme disease and others with similar clinical presentations. Using a high-throughput antibody-binding platform coupled with machine learning, the authors identified antigen-specific signatures capable of distinguishing true Lyme cases from similar clinical conditions, an advance that could improve diagnostic specificity in settings of clinical ambiguity, mainly in high burden countries.

The clinical interpretation of inflammatory biomarkers also gains complexity when stratified by patient characteristics. Feier et al. retrospectively analyzed systemic inflammatory features in individuals with acute appendicitis across age groups. Results demonstrated that markers such as neutrophil-to-lymphocyte ratio (NLR) and platelet-to-lymphocyte ratio (PLR) could be promised tools to preoperative stratification in acute appendicitis in different age groups. This highlights the importance of contextualizing even simple hematological parameters within the biological profile of the patient. Such stratified approaches, when incorporated into emergency and surgical settings, may sharpen risk prediction and guide timing and type of intervention.

In severe infections that could evolve with sepsis, mortality remains high despite protocolized care. In these settings system-level analyses can unearth mechanistic subtypes and prognostic axes, that can directly guide the clinical decision and intervention. Li et al. harnessed transcriptomic data from heterogeneous sepsis cohorts to identify and validate gene signatures associated with protein glycosylation. Results stratified patients according to risk groups with distinct prognosis. Pokharel et al., in parallel, focused on the mitochondrial fission protein Drp1 and identified a co-expression network of 7-gene signature associated with sepsis survival, suggesting that mitochondrial dynamics may represent an actionable axis in sepsis pathophysiology. Wang et al. further contributed mechanistic insight using experimental models of *Klebsiella pneumoniae* pneumosepsis. The study showed that elevated circulating levels of the polymeric immunoglobulin receptor (pIgR) exacerbated lung damage, positioning this traditionally “barrier-related” molecule as a potential target for mitigating alveolar injury in severe *Klebsiella pneumoniae* infections.

Beyond acute syndromes, systems immunology also allows for the detection of subtler, chronic immune alterations. Karisola et al. investigated individuals with post-COVID-19 condition, identifying sex-specific transcriptomic changes, particularly involving erythrocyte-related genes in males. These findings suggest lingering immune and hematological dysregulation even in the absence of evident active inflammation, with implications for understanding the persistence of symptoms in long COVID. Mao et al. addressed another vulnerable population by examining the impact of type 2 diabetes on pulmonary pathology in men co-infected with HIV and tuberculosis. The study demonstrated a strong association between diabetes and the presence of pulmonary cavitation, indicating that metabolic comorbidities modulate immune-mediated tissue damage and must be considered in radiological and therapeutic decision-making.

This intersection between immune characterization and rational intervention is also central to vaccine development, where systems immunology approaches are increasingly employed to map immunogenic targets. Suneesh et al. applied reverse vaccinology and immunoinformatic analyses to design multivalent mRNA constructs against herpes simplex virus type 2. By selecting conserved T and B cell epitopes and simulating their immunogenic properties in silico, the team laid the groundwork for future pre-clinical evaluation, advancing a pipeline that exemplifies rational antigen selection informed by systems biology.

Each contribution in this Topic leverages large-scale data and applies analytical tools ranging from statistical modelling to machine learning. What emerges is a portrait of systems immunology not as a theoretical framework, but as a practical toolkit for translational science. This toolkit enables the construction of biomarker panels tailored to context, the discovery of mechanistic pathways underlying disease progression, and the design of interventions - from diagnostic assays to therapeutic targets and immunization strategies - that are anchored in mechanistic insight rather than empirical generalization, with potential implications in patient care.

What binds this Research Topic is not a single disease or technology, but a shared philosophy: that complexity, if properly interrogated, can become a resource rather than a limitation. Systems immunology provides the means to interrogate this complexity, but its translational power depends on grounding in clinical relevance. Each study here exemplifies this principle: integrating omics with patient outcomes, repurposing known molecules into new functions, and refining the precision with which we diagnose, stratify, and treat infectious diseases. As these approaches continue to mature, they will increasingly define the interface between immunology and patient-centered care, closing the loop between bench and bedside.
